# Supplementary material for: HCMV promotes viral reactivation through the coordinated regulation of Notch signaling by UL8 and miR-UL36
Source: mBio. 2026 Feb 9;17(3):e03377-25. doi: 10.1128/mbio.03377-25 (PMC12977634; doi:10.1128/mbio.03377-25)
Supplement: Legends — Descriptions of supplemental figures and data set. [file mbio.03377-25-s0001.docx]

**Supplemental Figure 1: Treatment with CB-103 is not cytotoxic and does not impact lytic replication.** (A) CD34^+^ HPCs were incubated with 10 μM CB-103 or DMSO for 7 days. A colorimetric assay (WST-1 based, Roche) was used to determine cytotoxicity according to the manufacturer’s directions. Values are means±standard error (SD) (error bars). (B) NHDF cells were infected at an MOI of 0.05 and treated with 10 μM CB-103 or DMSO. Supernatants were harvested at the indicated times post-infection and titrated by TCID50.

**Supplemental Figure 2: Mutation of miR-UL36 does not affect pUL36 expression or virus replication.** (A) NHDF were infected with the indicated viruses at an MOI of 3. After 24 and 48 hours, protein lysates were harvested, and samples were immunoblotted using the indicated antibodies. (B) NHDF were infected with WT or ΔmiR-UL36 virus at an MOI of 3. At the indicated times post-infection, supernatant was harvested and titered on NHDF.

**Supplemental Figure 3: Characterization of viral mutants.** (A) Total DNA from primary CD34^+^ HPC cultures was extracted at 14 dpi and HCMV genomes were quantified using quantitative PCR with primers and probe specific for the *UL141* gene. Viral genomes were normalized to total cell number determined using human β-globin as a reference. Data shown is the mean value of three experiments and error bars represent standard deviation. (B) NHDF cells were infected with WT or UL8 Y305/314A mutant virus at an MOI of 0.05. Supernatants were harvested at the indicated times post-infection and titrated by TCID50. **(C)** Genome copy number as determined in (A). **(D)** NHDF cells were infected with WT or ΔmiR-UL36 mutant at an MOI of 0.05. Supernatants were harvested at the indicated times post-infection and titrated by TCID50.

**Dataset S1. Proteomic analysis of UL8 proximal proteins identified by TurboID-based proximity labelling.** The Excel file contains the complete mass spectrometry–based proteomics dataset and downstream analyses used to identify proteins in proximity to UL8 during HCMV infection using an *in vitro* TurboID labeling approach. UL8 was fused at its C-terminus to TurboID, and the recombinant virus was used to infect human fibroblasts. Upon biotin supplementation, TurboID-mediated biotinylation of neighboring proteins occurred in cultured cells. Biotinylated proteins were subsequently enriched and analyzed by LC–MS/MS. The file includes the following sheets:

- **Proteins:** Full list of identified proteins with associated metadata, including UniProt accession numbers, protein descriptions, peptide counts, sequence coverage, confidence scores, and false discovery rates (FDR).
- **normalized_data:** Quantitative protein abundance values after normalization across all samples.
- **log2_transformed+imputed data:** Normalized protein abundance values after log2 transformation and imputation of missing values, used for downstream statistical analyses.
- **UL8_proximal_proteins**: Subset of cellular and viral proteins significantly enriched in UL8–TurboID–expressing samples compared to controls, representing putative UL8-proximal proteins.
- **pathway_analysis:** Results of pathway and gene set enrichment analyses performed on the UL8-proximal proteins, including pathway names, gene counts, p-values, and FDR-adjusted significance values.
